# Phenolic compounds and antioxidant activities of moringa leaf processed by solid-state fermentation with *A.niger*

**DOI:** 10.3389/fnut.2026.1970171

**Published:** 2026-10-05

**Authors:** Mingjing Yang, Shuyu Pang, Lingyuan Luo, Xingru Zao, Xingfeng Deng, Yang Tian, Yongqiang Li

**Affiliations:** 1 College of Food Science and Technology, Yunnan Agricultural University, Kunming, China; 2 Miyi County Agriculture and Rural Bureau, Panzhihua, China; 3 Ganzi Tibetan Autonomous Prefecture Center for Disease Control and Prevention, Ganzi, China; 4 Engineering Research Center of Development and Utilization of Food and Drug Homologous Resources, Ministry of Education, Yunnan Agricultural University, Kunming, China

**Keywords:** *A.niger*, antioxidant activity, moringa leaf, phenolic compounds, solid-state fermentation

## Abstract

Moringa leaf (ML) is rich in phenolic acids, flavonoids, alkaloids, isothiocyanates, tannins and other phytochemicals with diverse biological effects. This study aimed to explore the effect of *A.niger* fermentation on phenolic compounds and antioxidant activities on the ML. The optimized fermentation conditions as follows: moisture content 60%, fermentation temperature 30°C, fermentation time 8 days and inoculation quantity 10^7^ spores/g fermentation sample. Filter paper enzyme (FPAse), carboxymethyl cellulase (CMCase), xylanase and β-glucosidase in ML increased significantly to 23.91 ± 0.85, 27.40 ± 1.12, 47.85 ± 0.91 and 2.45 ± 0.13 U/g DW respectively. Throughout 14 days of fermentation, a notable increase in total phenolic content (TPC), total flavonoid content (TFC) and antioxidant activity in soluble polyphenols (SPs) was observed. Specifically, TPC, TFC, DPPH radical scavenging activity (DRSA), trolox equivalent antioxidant capacity (TEAC), ferric reducing antioxidant power (FRAP) and hydrogen peroxide scavenging activity (HPSA) for ML were 146.74 ± 1.75 mg GAE/g DW, 2.12 ± 0.04 μmol CE/g DW, 32.56 ± 0.52 μmol TE/g D, 754.22 ± 3.24 μmol FE/g DW, 72.35 ± 0.25μmol FE/g DW, 105.79 ± 0.27μmol FE/g DW, respectively. In particular, the (-)-catechin, (-)-epicatechin were strongly positively correlated with the four antioxidant indexes. Moreover, fermentation with *A. niger* significantly increased the content of flavan-3-ols in ML. Furthermore, the inhibitory effects of α-amylase, α-glucosidase and lipase were significantly increased compared to unfermented moringa leaf (UF-ML) (*P* < 0.05). In summary, this study demonstrated that SSF was a new method for releasing polyphenols, which could provide a scientific basis for the further study of ML, as well as novel insights into the sustainable utilization of agricultural waste.

## 1 Introduction

*Moringa oleifera* (MO) belongs to the monogeneric genus Moringa of the Moringaceae family, is a perennial herb tree that widely grows in many countries in tropical and subtropical areas (1). Moringa conforms to the theory of food & medicine homology that it has a substance which integrates pharmacological and nutritional functions into one entity (2). Various parts of MO, including the leaves, roots, flowers, fruits, and pods, which can be used as an important source of nutrients and medicine in the food industry (3). However, ML was the most utilized part of MO plant (4). It contains multiple phytochemicals, including phenolic compounds, alkaloids, isothiocyanates and tannins (5). Currently, it has been approved as a new food resource by Chinese *Ministry of Health* in 2010.

Polyphenolic compounds are a class of natural secondary metabolites by shikimic acid, pentose phosphate, and phenylpropanol metabolic pathways that are widely found in plants (6). Due to its various bioactive activities, polyphenols are becoming more and more popular in functional foods and supplements in recent years. Extensive studies have confirmed that phenolic compounds have multiple biological activities such as antioxidant, anti-inflammatory, anti-diabetic, potential pharmacological, anti-asthmatic, anti-cancer activities and so on (7–11). The natural polyphenols are mainly separated into soluble polyphenols (SPs) and insoluble-bound polyphenols (IBPs) in plant (12). The soluble phenolic compounds can easily be extracted by using organic solvent, whereas the insoluble-bound polyphenolic compounds are covalently bond to cell wall macromlecules such as carbohydrate and proteins through ether ester, or glycosidic bounds so they are difficult to release. Hence several methods are used to extract the insoluble-bound polyphenolic compounds such as enzymatic treatment, chemical extraction, and microbial fermentation (13–15).

Currently, conventional chemical extraction methods for releasing polyphenolic compounds were alkaline hydrolysis and acid hydrolysis. Acidic hydrolysis mainly destroys the glycosidic bonds connected to polyphenols, but cannot destroy the ester and ether bonds connected to the matrix of insoluble bonded polyphenols (16). Meanwhile, high temperature acidic conditions may lead to the degradation of hydroxycinnamic acids such as *P*-coumaric acid, caffeic acid and ferulic acid (13). Compared with acid hydrolysis method, alkaline hydrolysis method is more effective in extraction, but alkaline hydrolysis method requires longer extraction time and complex processing process. Chemical extraction not only causes certain toxic effects on human body, but also has negative effects on the environment. enzymatic hydrolysis method by using various degradative enzymes, such as pectinases, cellulose, α-amylase, β-glucanases and xylanase, have been reported in several studies that their ability to cleavage the linkages between phenolic acids and sugars to release insoluble-bound polyphenolic compounds. This method is instability and high cost (17). Therefore, economically unfeasible. And more importantly, these methods are not sufficient to fully extract insoluble phenolic compounds, including those conjugated to peptides, cellulose, pectin, proteins, polysaccharides moieties or lignin. The microbial fermentation is a biotechnological processing method that is used to improve the release of phenolic components and the bioactivities of grain (18), beans (19), fruit (20) and so on. SSF, as a natural and economical method, has numerous advantages that minimal environmental pollution, inexpensive raw materials, and improve the nutritional value of food (21). The substrates must contain sufficient moisture to allow the microbial growth and metabolism. The selection of a suitable microorganism is one of the most important criteria in solid state fermentation bioprocessing. Numerous studies have discovered that SSF may enhance the bioactivity and bioavailability of natural polyphenols. During SSF, microorganisms produce carbohydrate-hydrolyzing enzymes, including β-glycosidase, α-glucanase, α-amylase, xylanase and cellulase, which destroyed the linkages between phenolic compounds and biological macromolecules to release the insoluble-bound phenolics, transforming them into soluble-free phenolics (22). *A. niger*, for example, is a useful common species of the genus Aspergillus which synthesize more than 19 types of enzymes (cellulase, pectinase, protease, etc.), This enzyme consortium to liberate the phenolic compounds in many SSF studies (23).

Therefore, this study aimed to investigate the influence of SSF by *A.niger* on phenolic contents and activity in ML. The dynamic changes of the content and antioxidant activity of phenolic fractions, hydrolase activity were analyzed during the fermentation process. And the correlation between hydrolase and polyphenol content, and the correlation between polyphenol content and antioxidant activity were initially discussed by using correlation heatmap analysis. Then the polyphenol compounds of SP and IBP from ML were identified before and after fermentation using high-performance liquid chromatography (HPLC). We expect that our study could provide a new idea for the biotransformation of the active ingredients in natural products and the scientific basis for the further development and utilization of ML.

## 2 Materials and methods

### 2.1 Materials, reagents and strains

ML obtained from Dehong Tianyou Technology Development Co. LTD, then were air dried, shredded through a disintegrator and a 60-mesh sieve to obtain the Moringa leaf powder, and stored at−20 °C in the dark for further use.

Standard phenolic compounds for high performance liquid chromatography (HPLC) analysis, including gallic acid, protocatechuic acid, (–)-epigallocatechin, (+)-catechin, p-hydroxybenzoic acid chlorogenic acid, vanillic acid, caffeic acid, vanillin, epicatechin, p-coumaric acid, ferulic acid, sinapic acid, vitexin, o-coumaric acid, ellagic acid, myricetin, trans-cinnamic acid, quercetin, kaempferol, apigenin and α-Amylase, α-glucosidase, lipase were purchased from Shanghai yuanye Bio-Technology Co., Ltd (Shanghai, China). Folin–Ciocalteu reagent, gallic acid, 2,2-diphenyl-1-picrylhydrazyl radical (DPPH), 2,2′-azinobis (3-ethylbenzothiazoline-6-sulfonic acid) (ABTS), 2,4,6-tripyridyl)-s-triazine (TPTZ), iron chloride (${\text{FeCl}}_{3}^{.}$6H_2_O), 2,2-diphenyl-2-picrylhydrazyl (DPPH), p-nitrophenyl-β-D-glucopyranoside (*p*-NPG), p-nitrophenol (*p*-NP) were purchased from Sigma-Aldrich (St. Louis, MO, USA). All other reagents were of analytical grade or above.

The strain *A.niger* (ATCC-6275) was purchased from the Guangdong Culture Collection Center.

### 2.2 Culture medium and SSF fermentation

The experiments were conducted in triplicate. Inoculating cultures of *A.niger* were revived by growing on PDA petridish and incubated at 30 °C for 10 days before use.

As for the SSF, ML was fermented according to methods described by (24, 25), the ML was mixed with distilled water in a ratio of 1:1 (w/w), and the mixture were sterilized in an autoclave at 121 °C for 30 min, After cooling to room temperature in Sterile microbiology chamber. The substrates were inoculated with spore suspension (2 × 10^7^ spores/g of solid). After being thoroughly mixing, the flasks with inoculated substrates were incubated at 30 °C for 14 days with a shaking speed of 120 r/min. During SSF, the samples were aseptically taken out after 0, 2, 4, 6, 8, 10, 12, 14 days for analysis and stored at−80 °C.

### 2.3 Optimization of optimum process

Based on the results of four factors experiments conditions included which are water content, fermentation time, fermentation temperature, inoculation quantity of *A. niger* while the response variable is Total Phenolic Content. Optimization by Box–Behnken response surface *design* consisting of 24 randomized experimental treatments with 5 center points was carried out. Factors and Levels in the Response Surface Analysis is shown in Table 1.

**Table 1:** Factors and levels of Box-Behnken experimental design.

Factors	levels		
	-1	0	1
A. fermentation temperature/°C	28	30	32
B. water content/%	55	60	65
C. fermentation time/d	6	8	10
D. inoculation quantity/10^n^ spores/g	6	7	8

### 2.4 Preparation of the soluble and insoluble-bound polyphenolic extracts

Soluble polyphenol fraction was extracted according to the methods described by (25). With some marginal modifications. The ML power was extracted twice with 20 ml of methanol solution (methanol: formic acid: H_2_O = 80: 0.1:19.9, v/v/v) at a radio of 1:10 (w/v) in triplicate. and performed in an ultrasonic bath for an hour. The extractions were repeated twice and centrifuged at 10,000 rpm for 10 min at 4 °C. the supernatants were collected and the above procedure was repeated once.

For the extraction of insoluble-bound Polyphenol, 40 ml NaOH (4 mol/L) was added into the residue after extraction of the soluble polyphenol, and hydrolyzed for 4 h under nitrogen gas. The mixture were acidified to pH 2 with 6 mol/L of HCl, and then centrifuged at 10,000 × g for 10 min at 4 °C. Then the supernatants were combined and extracted 3 times with an equal volume of diethyl ether and ethyl acetate (1:1, v/v), and the solvent was evaporated under vacuum at 45 °C to by using laboratory rotary evaporator.

### 2.5 Determination of TPC and TFC

The polyphenol content of SPs and insoluble-bound polyphenols (IBPs) in extracts from ML was determined by the Folin–Ciocalteu colorimetric method as described (25). Briefly, an appropriate amount of the sample was placed in a clean centrifugal tube, then 0.5 ml Folin–Ciocalteu reagent was added and mixed. Finally, 1 ml of 7.5% Na_2_CO_3_ solution was added and the volume was fixed to 10 ml with distilled water. After 2 h of incubation in the dark, the absorbance was determined at 760 nm. The TPC in samples was expressed as milligrams of gallic acid equivalents (GAE) per gram of the dry weight (DW) (mg GAE/g DW).

TFC was carried out by an aluminum chloride colorimetric assay according to the previously reported method (26). Standard curves were constructed with the catechin equivalent (CE) as standard and the absorbance was measured at 510 nm. The results obtained were expressed as μmol of CE per gram of the DW (μmol CE/g DW).

### 2.6 Preparation of crude enzyme solution

The crude enzyme solution from the fermented ML samples was extracted with 0.1mol/L, pH 4.8 citric acid buffer according to the ratio of solid to liquid at 1:10 (M/V), and the flasks were shaken at 28°C, 180 rpm, for 2 h. and then centrifuged at 4°C and 10,000 g for 15 min. The supernatant was for enzymes activity.

According to the previous method, the total cellulase activities including FPAse and CMCase activity were assayed with some modifications (25). Briefly, 1 ml crude enzyme solution and 1 ml of citric acid buffer with 50 ± 0.5 mg Whatman No.1 filter paper or 1 ml of 1% sodium carboxymethyl cellulose was added to start the reaction, and incubated at 45°C for 30 min. Then 1 ml of 3, 5-dinitrosalicylic acid (DNS) reagent was added into the mixture, and incubated at a boiling water bath for 5 min to termination reaction. The absorbance was measured at 540 nm.

The β-glucosidase activity was assayed based on the previous method with minor modifications (18). In brief, 1ml crude enzyme solution was mixed with 1 ml 5 mM p-NPG, and incubated at 37 °C for 10 min. Then 2 ml of 1 mol/L Na_2_CO_3_ solution was added to stop the reaction. The absorbance was measured at 400 nm.

The xylanase activity as evaluated based on the previous method with minor modifications (25). The reaction system included 1ml crude enzyme solution and 1 ml of 2% xylan (w/v, in 0.1mol/L citric acid buffer, pH 4.8) and incubated at 45°C for 30 min. Then 1 ml of 3, 5-dinitrosalicylic acid (DNS) reagent was added into the mixture, and incubated at a boiling water bath for 5 min to termination reaction. The absorbance was measured at 540 nm.

One unit (U) of CMCase (FPase/β-glucosidase /xylanase) activity was defined as the quantity of enzyme required to release one micromole of glucose (glucose/xylose/p-nitro phenol) per minute under the assay conditions used, and the results were expressed by U/g DW.

### 2.7 Determination of *in vitro* antioxidant activity

#### 2.7.1 DRSA assay

The DRSA of non- or fermented from ML sample was evaluated according to the previous method, with slight modifications (12, 25). In Brief, 4ml 79μmol/L DPPH-methanol solution was mixed with 1ml the phenolic extract. Then the mixture incubated in the darkness for 10 min at ambient temperature, and the absorbance was read at 517 nm. The DRSA was established according to the following formula:


$$\begin{array}{c} \text{DRSA}\left(\text{\%}\right)=\left[\left(\text{A}-\text{Ab}\right)-\left(\text{C}-\text{Cb}\right)\right]/\left(\text{A}-\text{Ab})\times 100\text{\%}\right] \end{array}$$ (1)

where A is absorbance of the DPPH solution, Ab is absorbance of the methanol, C is absorbance of the DPPH solution with the sample, and Cb is absorbance of the methanol with the sample. The standard curve was prepared using Trolox and expressed as μmol of TE per gram of dry weight (μmol TE/g DW).

#### 2.7.2 TEAC assay

The TEAC was measured using the method of previously reported, with minor modifications (12, 27). In Brief, The ABTS working solution was made by mixing ABTS solution 7 mmol/L with potassium persulfate 2.45 mmol/L (1:1 v/v). The reaction solution containing 100 μl of phenolic extract and 3.8 ml of ABTS working solution. and the absorbance of the samples was read at 734 nm. The TEAC was established using the following formula:


$$\begin{array}{c} \text{TEAC}\left(\text{\%}\right)=\left[\left(\text{A}-\text{Ab}\right)-\left(\text{C}-\text{Cb}\right)\right]/\left(\text{A}-\text{Ab})\times 100\text{\%}\right] \end{array}$$ (2)

where A is absorbance of the ABTS + working solution, Ab is absorbance of the methanol, C is absorbance of the sample solution with the sample with the ABTS + working solution, and Cb is absorbance of the methanol with the sample. The standard curve was made using Trolox and expressed as μmol of TE per gram of dry weight (μmol TE/g DW).

#### 2.7.3 FRAP assay

The FRAP was measured using the method of previously reported, with slight modifications (12, 25). In Brief, The FRAP working solution was made by mixing the acetate buffer (300 mmol/L, pH 3.6), FeCl_3_ solution (20 mmol/L), and TPTZ (10 mmol/L) in a volume ratio of 1:1:1. The reaction solution containing 100 μl of phenolic extract and 3ml of FRAP working solution and incubated at 37°C for 4 min. After the absorbance of the samples was read at 734 nm. Ferrous sulfate was used as a reference standard, and the FRAP was expressed as μmol of Fe^2+^ equivalents (FE) per gram of dry weight (μmol FE/g DW).

#### 2.7.4 HPSA assay

The HPSA was measured using the method of previously reported, with slight modifications (12, 25). Briefly, the phenolic extract (600 μl) was mixed with 0.9 ml H_2_O_2_ (400 mmol/L) and 1.5 ml sodium phosphate buffer (45 mmol/L, pH 7.4). The reaction mixture was incubated for 40 min in the dark and then its absorbance was measured at 230 nm. The HPSA was calculated according to the following formula:


$$\begin{array}{c} \text{HPSA}\left(\text{\%}\right)=\left[\left(\text{A}-\text{Ab}\right)-\left(\text{C}-\text{Cb}\right)\right]/\left(\text{A}-\text{Ab})\times 100\text{\%}\right] \end{array}$$ (3)

where A is absorbance of the H_2_O_2_ with the PBS, Ab is absorbance of the PBS, C is absorbance of the H_2_O_2_ and the sample with the PBS and Cb is absorbance of the sample and the PBS. The Trolox was used to prepare the standard curve, the HPSA was expressed as μmol of TE per gram of dry weight (μmol TE/g DW).

### 2.8 Determinations of inhibitory activities of α-Amylase and α-Glucosidase

#### 2.8.1 α-Amylase inhibition

The α-amylase inhibition ability was assessed based on the previous method with minor modification (28). Initially, 500 μl of ML extract with different concentrations (12.5–1,000 μg GAE/ml) was mixed with 500 μl 13 U/ml porcine pancreatic α-amylase prepared in PBS buffer (0.1 mol/L, pH 6.9), and incubated for 10 min at 37 °C. Then, 500 μl of gelatinized starch solution (1%, w/v) was added into the mixture, and incubated for another 10 min at 37 °C. Followed the addition of 1 ml of 3, 5-dinitrosalicylic acid (DNS) reagent was added into the mixture, and incubated at a boiling water bath for 5 min. Finally, the absorbance of the mixture was measured at 540 nm. Acarbose was used as positive control and the obtained inhibitory activities were expressed as the IC50 value (ug/ml). The α-amylase inhibitory activity was calculated according to the following formula:


$$\begin{array}{c} \text{α}-\text{Amylase inhibition}\left(\text{\%}\right)=\left(1-\frac{\text{As}-\text{A}{\text{s}}^{\text{′}}}{\text{Ac}-\text{A}{\text{c}}^{\text{′}}}\right)\times 100\text{\%} \end{array}$$ (4)

Where As is the absorbance of sample, enzyme, substrate and DNS, As' is the absorbance of sample, substrate and DNS, Ac is the absorbance of buffer solution, enzyme, substrate and DNS, Ac′ is the absorbance of buffer, substrate and DNS.

#### 2.8.2 α-Glucosidase inhibition

According to the previous method with some modifications, the α-glucosidase inhibition ability was measured (29). Briefly, 50 μl of the different concentrations (12.5–1,000 μg GAE/ml) of ML extract and 50 μl of 1U/ml α-glucosidase prepared in phosphate buffer (0.1 mol/L, pH 6.9) were mixed in 10 ml tube. After incubation at 37°C for 10 min, 50μl of 5 mmol/L pNPG (solved in phosphate buffer 0.1 mol/L, pH 6.9) was added and incubated at 37°C for 10 min. Finally, 2 ml of 0.1 M Na_2_CO_3_ solution was added to terminate the reaction. The absorbance was measured at 405 nm. Acarbose was used as positive control and the obtained inhibitory activities were expressed as the IC50 value (ug/ml). The results were calculated according to the following formula:


$$\begin{array}{c} \text{α}-\text{Glucosidase inhibition}\left(\text{\%}\right)=\left(1-\frac{\text{As}-\text{A}{\text{s}}^{\text{′}}}{\text{Ac}-\text{A}{\text{c}}^{\text{′}}}\right)\times 100\text{\%} \end{array}$$ (5)

Where As is the absorbance of sample, enzyme, pNPG and Na_2_CO_3_, As' is the absorbance of sample, pNPG and Na_2_CO_3_, Ac is the absorbance of buffer solution, enzyme, substrate, pNPG and Na_2_CO_3_, Ac′ is the absorbance of buffer, pNPG and DNS.

#### 2.8.3 Lipase inhibition

The method for measuring pancreatic lipase inhibition was done through following previously method with slight modifications (30). First, 40 μl 0.1M phosphate buffer (pH 6.9) was added into 10 ml tube. Then, 40 μl of ML extract at the concentrations of 12.5-1,000 μg GAE/ml was mixed with 40μl of pancreatic lipase solution (2.5 mg/ml) dissolved with phosphate buffer, and the mixture was incubated at 37°C for 15 min. After incubation, 20 μl of pNPP (0.1M) dissolved with isopropanol was added to each tube and incubated for another 15 min at 37 °C. At the end of the incubation, 100 μl anhydrous ethanol solution was added to stop the reaction. Finally, the absorbance was recorded at 405 nm, and orlistat was used as positive control and the obtained inhibitory activities were expressed as the IC50 value (ug/ml). The lipase inhibition was calculated according to the following formula:


$$\begin{array}{c} \text{Lipase inhibition}\left(\text{\%}\right)=\left(1-\frac{\text{As}-\text{A}{\text{s}}^{\text{′}}}{\text{Ac}-\text{A}{\text{c}}^{\text{′}}}\right)\times 100\text{\%} \end{array}$$ (6)

Where as is the absorbance of sample, enzyme and pNPP, As' is the absorbance of sample and pNPP, Ac is the absorbance of buffer, enzyme and pNPP, Ac′ is the absorbance of buffer and pNPP.

### 2.9 HPLC analysis

The main phenolic compounds of SPs and IBP extracts of 8 d fermentation ML (F-ML), and unfermented moringa leaf (UF-ML) were analyzed by HPLC as previously reported (25). An Agilent 1,100 HPLC system (Agilent Technologies, Palo Alto, Calif, GER) equipped with a G7114A diode array detector (DAD). The separation was carried out on an Agilent Poroshell120EC-C18 column (150 × 4.6 mm, 5 μm). The mobile phases consisted of methanol (A) and water with 0.1% formic acid (B) applied at a flow rate of 0.8 ml/min. The gradient elution was programmed as follows: 0–7 min, 15% A ~ 85% B; 7–13 min, 20% A ~ 80% B; 13–20 min, 20% A ~ 80% B; 20–30min, 30% A ~ 70% B; 30–35min, 65% A ~ 35% B. The operating temperature of the column was maintained at 30 °C, the flow rate of 0.8 ml/min. and the volume of injection was 2 μl. The detect wavelength was performed at 280 nm. Identification and quantification of the 21 phenolic compounds were based on of their retention time and characteristic absorption spectrum from the DAD with those of their authentic standards.

### 2.10 Statistical analyses

All the results were expressed as mean ± standard deviation (SD), and measurements were characterized the independent experiments in triplicate. Correlations of the carbohydrate-hydrolysing enzymes activities and the phenolic content, the phenolic content and antioxidant activity were measured using Pearson's correlation. The significant associations were determined using the one-way analysis of variance (ANOVA) followed by Duncan's multiple tests. A probability value of *p* < 0.05 was considered statistically significant.

## 3 Results and discussion

### 3.1 Fermentation conditions of model optimization

Based on the experimental results of single-factor, RSM was employed to optimize the parameters of SSF. It was indicate from the Table 2 that a multinomial linear regression analysis of the experimental results was carried out by Design-Expert 8.0.6 software, and the quadratic polynomial regression model equation was established:


$$\begin{array}{l} Y=141.40+2.76A-6.79B-3.88C-5.19D-4.15AB\\ \;-8.21AC+4.38AD+9.74BC-9.91BD+1.55CD\\ \;-14.81A^{2}-20.90B^{2}-19.49C^{2}-21.13D^{2} \end{array}$$

Y, the total phenol content; A, the fermentation temperature (°C); B, the water content (%); C, the fermentation time; D, the inoculation quantity.

**Table 2:** Box–Behnken design and response values of RSM.

Runs	A	B	C	D	Yield
	Fermentation temperature	Water content	Fermentation time	Inoculation quantity	(mg GAE/g DW)
1	32	60	6	7	120.20
2	30	65	6	7	91.31
3	30	60	6	6	110.11
4	30	60	6	8	89.18
5	28	60	6	7	112.28
6	30	55	6	7	118.05
7	28	55	8	7	102.56
8	28	60	8	6	98.10
9	32	60	8	6	105.21
10	32	65	8	7	97.16
11	30	65	8	8	75.49
12	30	60	8	7	141.66
13	30	55	8	8	114.16
14	30	60	8	7	148.44
15	32	60	8	8	117.36
16	28	65	8	7	96.24
17	30	60	8	7	140.29
18	30	55	8	6	111.06
19	28	60	8	8	92.71
20	32	55	8	7	120.07
21	30	60	8	7	141.08
22	30	65	8	6	112.02
23	30	60	8	7	135.54
24	32	60	10	7	93.12
25	30	60	10	8	91.21
26	30	55	10	7	87.01
27	28	60	10	7	118.05
28	30	65	10	7	99.24
29	30	60	10	6	105.93

The regression model is highly significant according to results of ANOVA for model testing in Table 3 that the F value was 8.59, and the *P* < 0.0001. In addition, The lack of fit was not significant (*P* > 0.05), and the determination coefficient *R*^2^ value of the regression model is 0.9543, which was closed to 1, which indicated that the fitting model had a good fitting degree can accurately predict the experimental result (31).

**Table 3:** Analysis of variance of regression model.

Source	Sum of squares	df	Mean square	F value	P	Significance
Model	8,579.63	14	612.83	8.59	0.0001	^***^
A–fermentation temperature	91.64	1	91.64	1.29	0.2760	
B–water conten	552.92	1	552.92	7.75	0.0146	^*^
C–fermentation time	180.75	1	180.75	2.53	0.1337	
D–inoculation quantity	323.54	1	323.54	4.54	0.0514	
AB	68.82	1	68.82	0.9650	0.3426	
AC	269.80	1	269.80	3.78	0.0721	
AD	76.88	1	76.88	1.08	0.3167	
BC	379.76	1	379.76	5.33	0.0368	^*^
BD	392.71	1	392.71	5.51	0.0342	^*^
CD	9.65	1	9.65	0.1354	0.7184	
A^2^	1,423.11	1	1,423.11	19.96	0.0005	^***^
B^2^	2,833.30	1	2,833.30	39.73	< 0.0001	^***^
C^2^	2,462.76	1	2,462.76	34.54	< 0.0001	^***^
D^2^	2,895.05	1	2,895.05	40.60	< 0.0001	^***^
Residual	998.34	14	71.31			
Lack of fit	913.02	10	91.30	4.28	0.0869	not significant
Pure error	85.32	4	21.33			
Cor total	9,577.97	28				
Adj R–squared	0.79					

^*^P < 0.05. ^**^P < 0.01. ^***^P < 0.001.

The adjusted decision coefficient of the model (*R*^2^ *Adj* =0.908) closed to *R*^2^ illuminated that the model was excellent, and the coefficient of variation (CV= 7.69% < 10%), which showed that the experimental values was reliable and repeatable (32). Meanwhile, the response surface and contour maps of the variables and their interactions were shown in Figure 1. According to the value of F of the factors could confirm the effect of water content had significant deviation on the response variables of TPC (*P* < 0.05), followed by inoculation quantity (*P* < 0.05), fermentation time (*P* < 0.05), and fermentation temperature (*P* < 0.05). The optimal fermentation parameters were simulated as follows: water content 58.97%, fermentation time 7.81days, inoculation quantity 1x10^6.92^ CFU/g FS. TPC reached 146 ± 1.45 mg GAE/g DW under the conditions mentioned above. Significantly, for the sake of Considering the accuracy of the model analysis and the feasibility of operation, the fermentation conditions were adjusted as follows: fermentation temperature 30 °C, fermentation time 8 days, water content 60%, inoculation quantity 1x10^7^ CFU/g FS. The average values performed from three parallel verification experiments confirmed the reliability of the model. The results demonstrated that the experimental values were in good agreement with the predicted values, and the RSM model was applicable.

**Figure 1:**
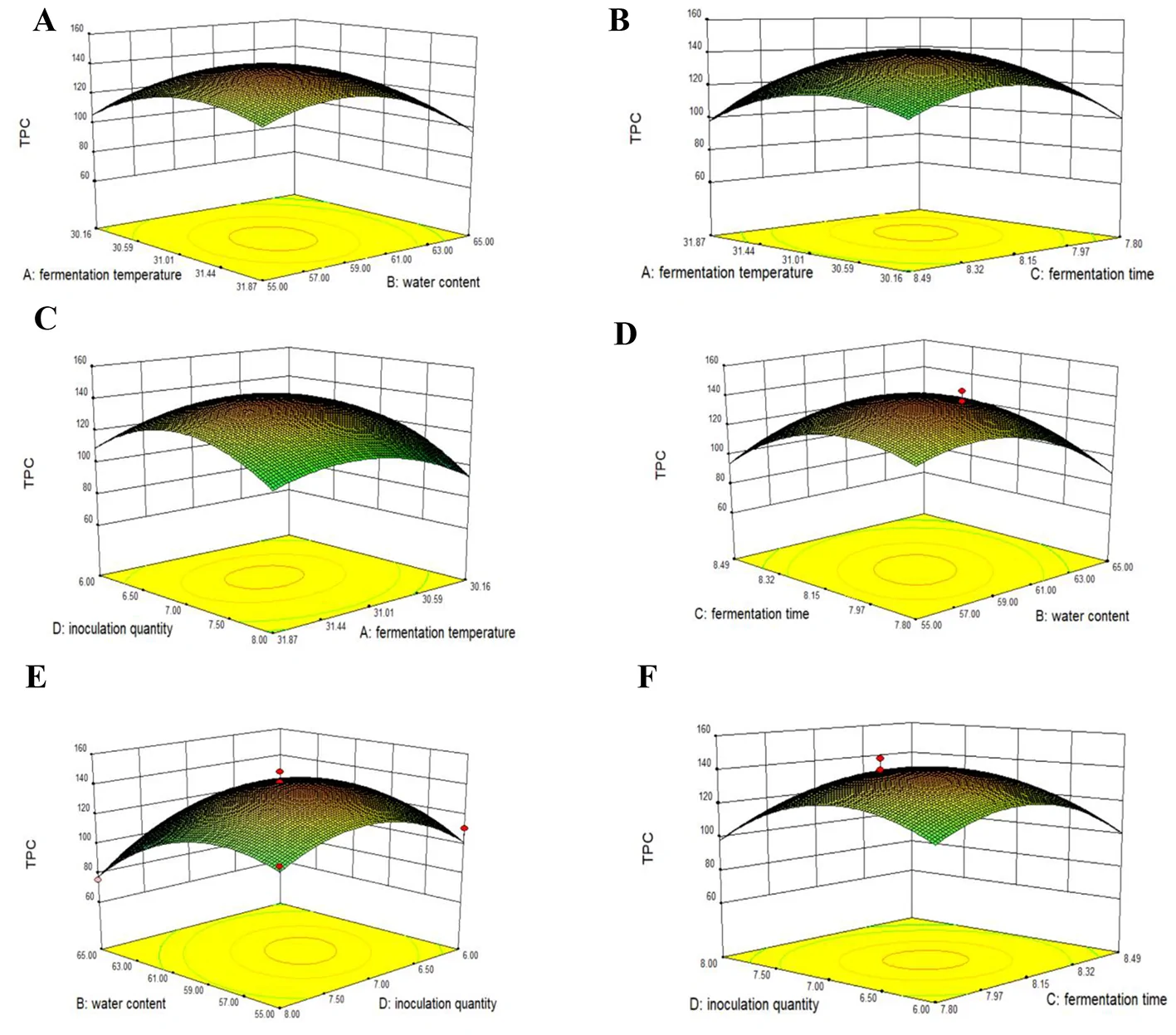
Response surface plots showing the effects of fermentation parameters on total phenolic content (TPC). **(A)** fermentation temperature and water content; **(B)** fermentation temperature and fermentation time; **(C)** fermentation temperature and inoculation quantity; **(D)** fermentation time and water content; **(E)** water content and inoculation quantity; and **(F)** inoculation quantity and fermentation time.

### 3.2 Carbohydrate-hydrolysing enzymes activities

Some hydrolytic enzymes produced by fungi that included cellulase, xylanase, and β-glucosidase were effective in releasing of IBP (33). To investigate the changes of carbohydrate hydrolyzing enzymse activities produced by *A. niger* during the fermentation process of ML. As shown in Figure 2A, the xylanase activities were much higher than CMCase, β-glucosidase and FPase activities during the whole fermentation process. The activities of the xylanase, CMCase, β-glucosidase and FPase were in a low state at the initial phases of fermentation. It showed a trend of first increase and then decrease for the activities of the four enzymes with increasing length of fermentation time. The maximum activities of xylanase, CMCase, and β-glucosidase reached on the 8th day of fermentation. The value of activities was 47.85 ± 0.91, 27.40 ± 1.12 and 2.45 ± 0.13 U/g DW, respectively. While the FPase activity peaked on the 6^th^ day was 23.91 ± 0.85 U/g DW. It was generally recognized that xylanase could interrupt the cell wall of IBPs and resulted in fully decomposition of xylan, which was the most plentiful component in hemicellulose (18). The results were similar to previous studies in that *A. niger* were highly productive of xylanase activity, which seemed to facilitate the decomposition of hemicellulose (34)

**Figure 2:**
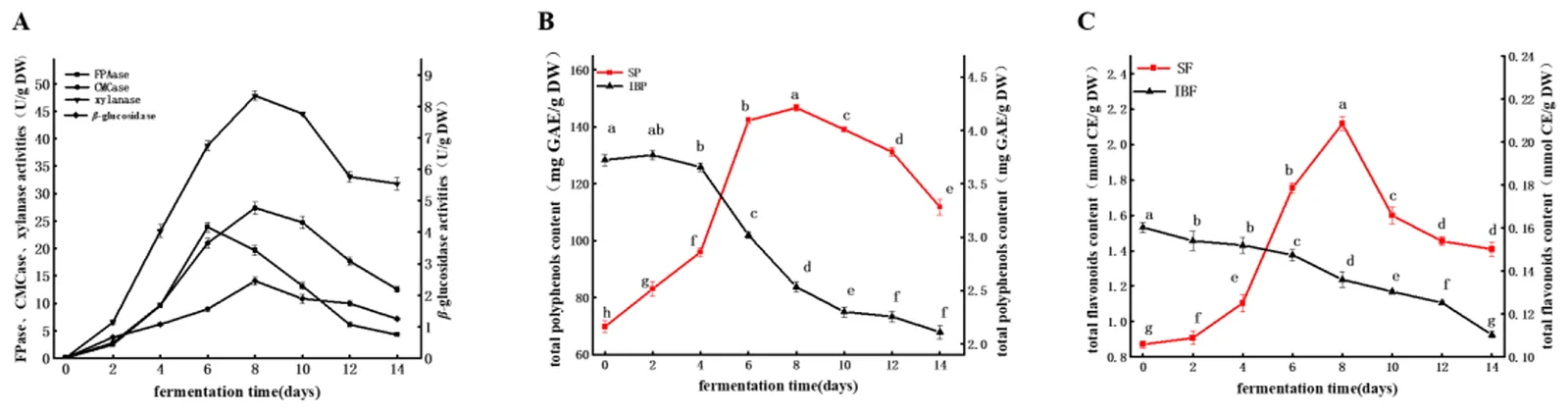
The dynamic Changes of **(A)** hydrolase activity, **(B)** total polyphenols content (TPC) and **(C)** total flavonoids content (TFC) during fermentation of ML. FPAse, filter paper enzyme; CMCase, carboxymethyl cellulase; SP, soluble polyphenol; IBP, insoluble-bound polyphenol; SF, soluble flavonoid; IBF, insoluble-bound flavonoid. Values represent mean ± SD (*n* = 3). Different letters within each treatment denote a significant difference (*P* < 0.05).

### 3.3 Effect of SSF on TPC and TFC

In recent years, microbial fermentation is an alternative method to enhance bioactive compound release from plant sources (13, 35). As shown in the TPC (Figure 2B) and TFC (Figure 2C), SPs fraction increased firstly and then decreased with the increase of fermentation time, and reached their highest content on the 8th day 146.74 ± 1.75 mg GAE/g DW, 2.12 ± 0.04 μmol CE/g DW, respectively. In contrast, the IBPs content decreased remarkably. During microbial fermentation, solid fermentation can simulate a natural environment conducive to the growth of microorganisms, so the selection of the right strain is crucial, most fungi and bacteria have the advantages of strong adaptability and can penetrate the solid matrix, etc., in the fermentation process can produce a variety of hydrolases, is conducive to promoting the release of insoluble bonded polyphenols, can increase the content of polyphenols, improve the biological activity of polyphenols (36).

However, the structure of polyphenolic could be damaged by a few hydrolytic enzymes made by fungi, such as xylanase, cellulase, and -glucosidase. Previous studies have demonstrated that *A. niger* could produce cellulase and xylanase to release phenolic compounds conjugated with xylan, cellulose, or lignin (37). In order to elucidate the effects of the CMCase, xylanase, FPase and β-glucosidase activities on releasing content of SP and IBP, the correlation matrix analysis were further performed by means of the correlation heatmap and are displayed in Figure 3A. Herein, the correlation coefficients *R*^2^ of CMCase, xylanase, β-glucosidase and FPase enzyme to SP content were 0.97, 0.96, 0.93 and 0.83, respectively. and their *R*^2^ values were all greater than 0.7, showing extremely significant positive correlation (*P* < 0.05). This data suggested that the CMCase, xylanase, β-glucosidase and FPAas produced by the *A.niger* is involved in the breakdown of the ML cell walls and the subsequent liberation of the phenolic compounds during the fermentation. However, no significant correlation was observed between the activities of carbohydrate-hydrolyzing enzymes and IBP content. This may be because hydrolases produced by microorganisms during fermentation facilitate the release of IBPs from the food matrix and promote their conversion into soluble phenolic compounds. The enzymatic action of xylanase and β-glucosidase hydrolyzes glucan and xylan into glucose and xylose. These sugars serve as nutrients to support the growth and development of mycelium, leading to increased levels of bioactive substances, including polysaccharides, triterpenes, total phenols, and flavonoids (38). This is similar to the content of the report by Zhao et al. (39), indicate that hydrolytic enzymes (e.g., cellulase and xylanase) produced via SSF of *Pleurotus ostreatus* facilitate the disruption of mulberry leaf cell structures, thereby improving the composition and content of polyphenols and flavonoids. Fermentation of guava leaves by *M. anka* GIM 3.592 and *S. cerevisiae* GIM 2.139 promoted the release of chlorogenic acid, rutin and quercetin (40).

**Figure 3:**
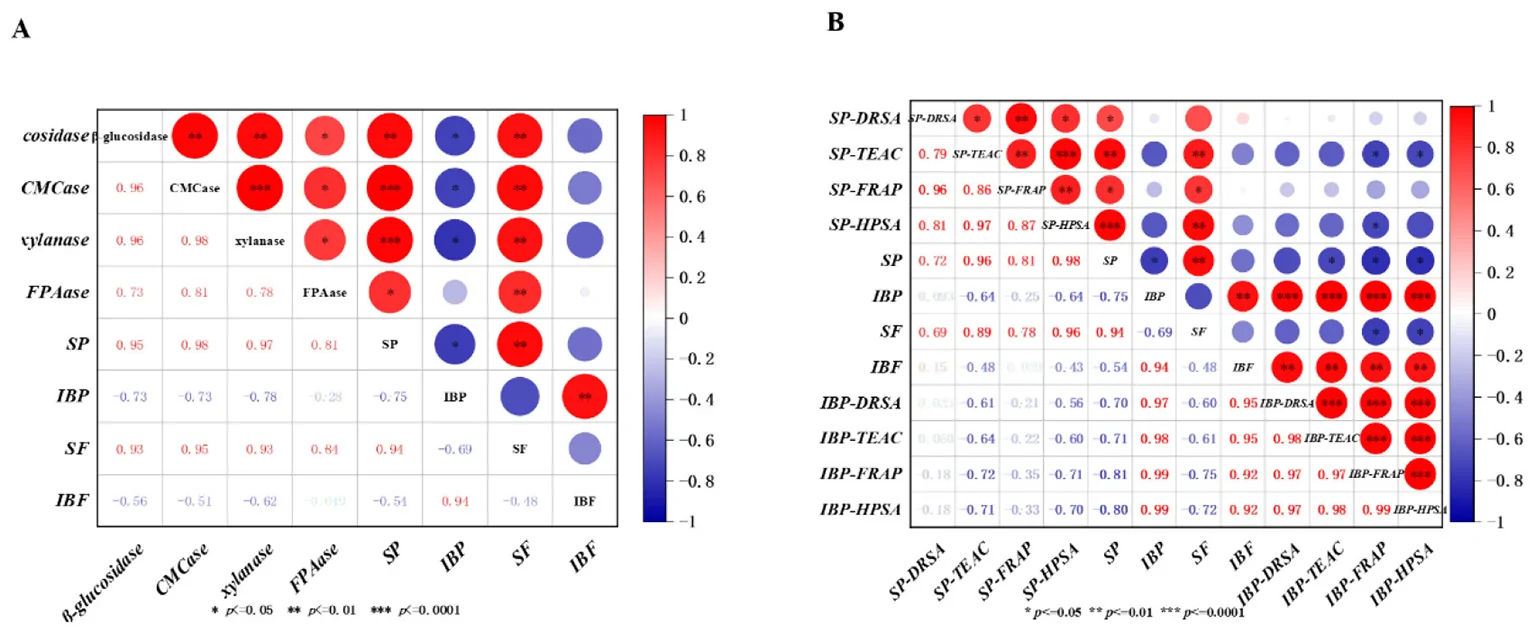
Correlation analysis in ML extracts. **A**. Phenolic contents and carbohydrate-hydrolysing enzymes activities; **B**. polyphenols content and antioxidant activities C. phenolic compounds and antioxidant activities. The values represent mean ± SD (*n* = 3). *, *P* < 0.05; **, *P* < 0.01; ***, *P* < 0.001.

### 3.4 Phenolic compounds by HPLC analysis

HPLC systems coupled with a PDA detector was performed to the qualitatively and quantitatively analyze the changes of 21 phenolic compounds from SP and IBP of ML system (Figure 4). As shown in Table 4, a total of 19 compounds were identified and categorized into three classes: hydroxybenzoic acids, hydroxycinnamic acids, and flavonoids. Following fermentation with *A. niger*, the content of soluble polyphenols in ML increased significantly. Specifically, the levels of p-hydroxybenzoic acid, p-coumaric acid, trans-cinnamic acid, (-)-epicatechin, vitexin, apigenin, and (+)-catechin increased by 1.32-, 1.37-, 43-, 1.65-, 1.4-, 1.48-, and 1.98-fold, respectively. This can be attributed to the facilitated release of insoluble bound polyphenols during fermentation, which thereby increased the content of soluble free polyphenols. Although 21 phenolic compounds were quantified by HPLC, these compounds represented only a fraction of the total phenolic constituents in fermented Moringa leaf extracts. The TPC values compared with the quantified HPLC phenolics may be attributed to the presence of unidentified phenolic derivatives, other reducing substances, and newly transformed phenolic compounds generated during microbial fermentation that were not included in the current HPLC standard. It was interesting that vanillin was only found in F-ML, which might be attributed to the action of microorganisms or hydrolytic enzymes that promoted the conversion of the hydroxybenzoic acids pathway. As shown in Figure 5, it is worth noting that the flavan-3-ol biosynthetic pathway (e.g., (-)-epicatechin, (+)-catechin) significantly upregulated in F-ML. As a major subclass of flavonoids, flavan-3-ols possess a variety of bioactive phytochemicals and naturally exist in tea, cocoa, grapes, apples, strawberries and persimmons (41, 42). For example, (+)-catechin is capable of stimulating insulin secretion in pancreatic β-cells, demonstrating their potential as candidates for diabetes treatment (43).

**Figure 4:**
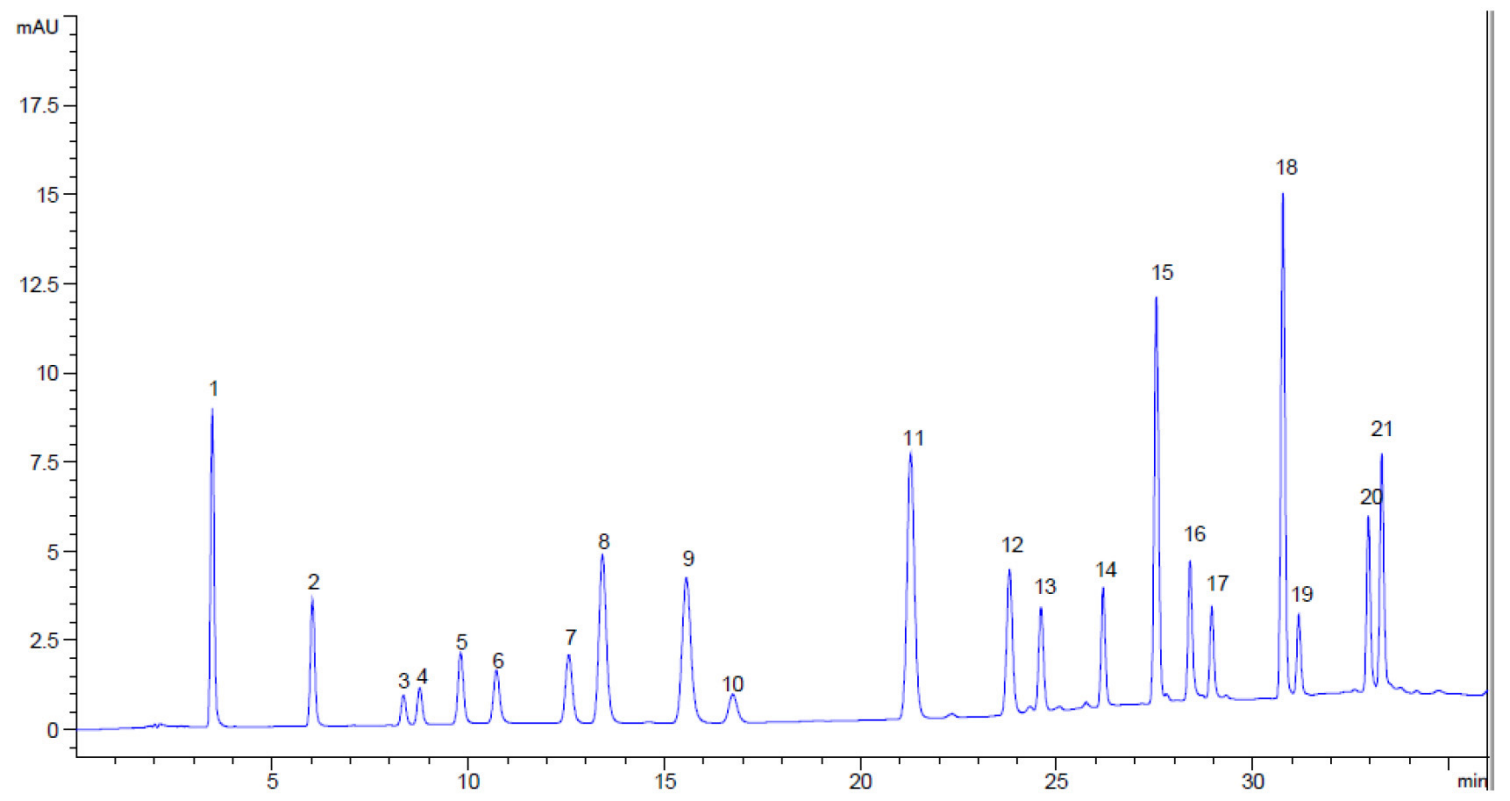
Chromatogram of phenolic compounds standard mixture. HPLC chromatograms of phenolic fractions in ML. 1. gallic acid; 2. protocatechuic acid; 3. (–)-Epigallocatechin; 4. (+)-catechin; 5. p-Hydroxybenzoic acid; 6. Chlorogenic acid; 7. Vanillic acid; 8. caffeic acid; 9. Vanillin; 10. Epicatechin; 11. p-coumaric acid; 12. ferulic acid;13. Sinapic acid; 14. Vitexin; 15. o-Coumaric acid; 16. ellagic acid; 17. myricetin; 18. trans-Cinnamic acid; 19. quercetin; 20. kaempferol; 21. apigenin.

**Table 4:** Content of soluble and insoluble bound phenolic compounds in ML.

Compounds	Fermentation system			
	UF–ML		F–ML	
	SPs	IBPs	SPs	IBPs
Protocatechuic acid	131.61 ± 6.62^Bf^	10.84 ± 0.16^Bb^	127.16 ± 8.77^Ae^	11.29 ± 1.01^B^f
p–Hydroxybenzoic acid	100.05 ± 6.15^Bg^	ND	132.45 ± 8.39^Ae^	16.96 ± 0.16^Cd^
Gallic acid	ND	2.55 ± 0.13^b^	ND	ND
Vanillin	ND	3.19 ± 0.75^b^	65.56 ± 2.04^f^	ND
Chlorogenic acid	560.98 ± 20.52^Ac^	19.54 ± 1.93^Cb^	138.11 ± 57.08^Be^	ND
Caffeic acid	13.48 ± 1.63^Ch^	212.18 ± 3.35^Aa^	ND	144.10 ± 0.17^Ba^
p–coumaric acid	182.44 ± 2.87^Be^	25.18 ± 0.31^Cb^	251.19 ± 2.15^Ad^	26.59 ± 0.04^Db^
Trans–Cinnamic acid	5.43 ± 1.13^Bh^	1.96 ± 0.04^Bb^	234.59 ± 5.36^Ad^	1.49 ± 0.01^Bg^
Ferulic acid	ND	21.43 ± 5.98^Ab^	ND	20.07 ± 0.35^Ac^
o–Coumaric acid	ND	ND	ND	ND
Sinapic acid	ND	ND	ND	ND
(-)–Epicatechin	596.28 ± 39.04^Bb^	ND	983.94 ± 11.08^Ab^	ND
Vitexin	160.74 ± 9.56B^Bef^	21.12 ± 0.35^Cb^	225.15 ± 7.27^Ad^	14.12 ± 0.07^Ce^
Quercetin	462.59 ± 41.34^Ad^	26.27 ± 7.73^Cb^	318.15 ± 23.66^Bc^	ND
Kaempferol	142.36 ± 13.85^Af^	0.42 ± 0.00^Cb^	126.15 ± 6.13^Be^	ND
Apigenin	23.22 ± 0.21^Bh^	ND	34.43 ± 2.49^Af^	ND
(+)–catechin	641.78 ± 3.79^Ba^	ND	1273.46 ± 37.53^Aa^	ND
(–)–Epigallocatechin	ND	ND	ND	ND
Myricetin	ND	ND	ND	0.67 ± 0.00^h^
Ellagic acid	ND	ND	ND	1.06 ± 0.08^gh^

Values represent the mean ± SD (*n* = 3). Different lowercase letters indicate significant differences within the column, different capital letters indicate significant differences within the row, while the same letter indicates no significant differences (*P* < 0.05). ND: not detected; UF-ML denotes unfermented moringa leaf, F-ML denotes fermented moringa leaf, SPs denotes soluble polyphenols, and IBPs denotes insoluble-bound polyphenols.

**Figure 5:**
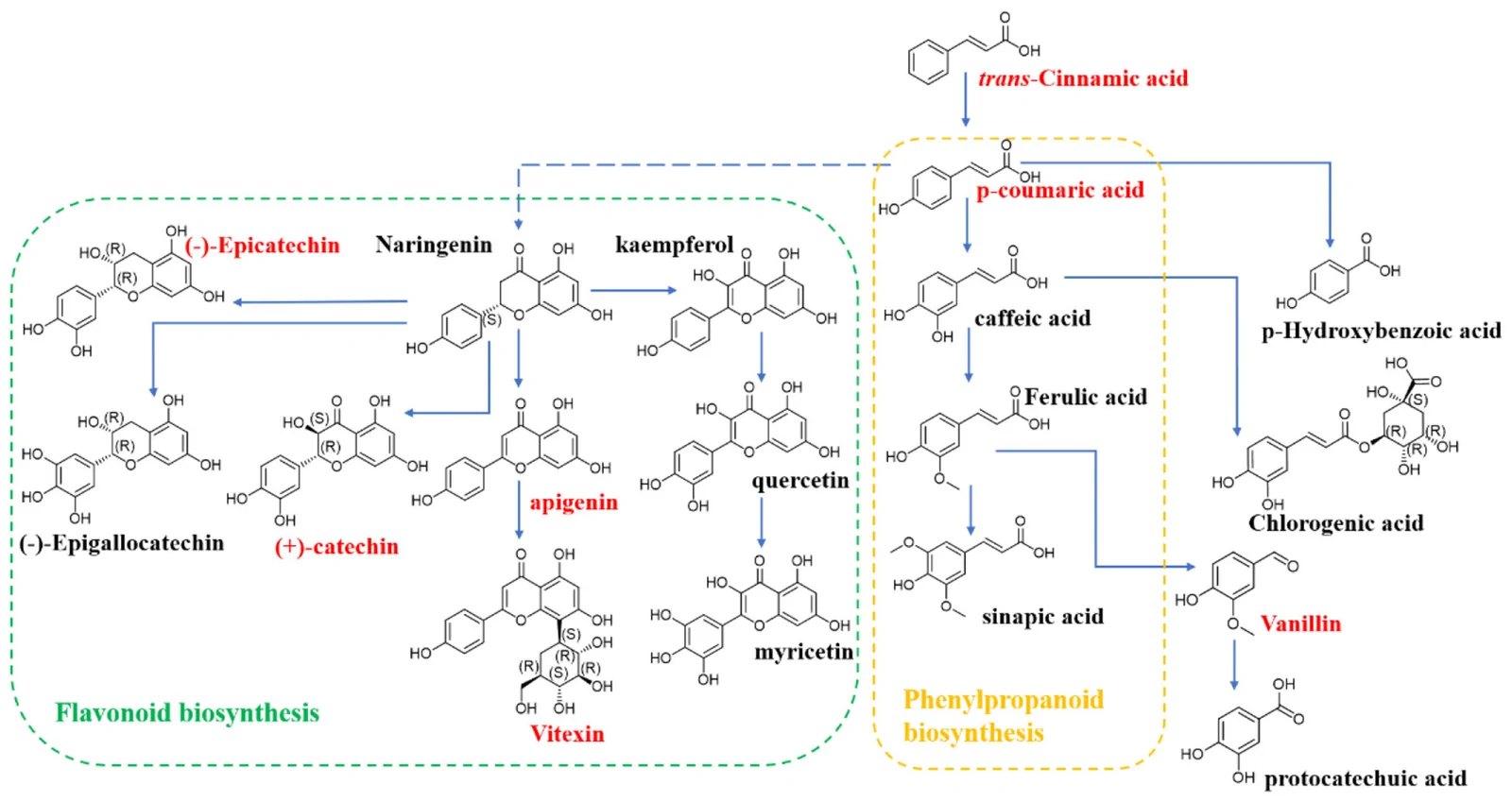
Biotransformation pathways of SPs in ML involves hydroxybenzoic acids, hydroxycinnamic acids and flavonoids. Red represents the elevated compound and the dotted line represents an unquantified compound.

### 3.5 Effect of SSF on antioxidant activity of ML

The antioxidant capacity of the SP and IBP of ML extracts during the SSF were evaluated using the DRSA, TEAC, FRAP and HPSA. The results indicated that a trend of first increase and then decrease for DRSA, TEAC, FRAP and HPSA of the soluble polyphenol. As shown in Figure 6, the DRSA of soluble polyphenols in ML reached the highest, which was 32.56 ± 0.52 μmol TE/g DW On the 6th day of fermentation; The maximum value of FRAP, TEAC and HPSA on the 8th day of fermentation was 72.35 ± 0.25, 754.22 ± 3.24 and 105.79 ± 0.27μmol FE/g DW, respectively. Compared with 0 days of fermentation, DRSA, FRAP, TEAC and HPSA increased by 73.6% 69.7% 86.6% 86.9%, respectively. However, the anti-oxidant capacity of insoluble-bound polyphenol is a slow downward trend. This may be because microorganisms release insoluble bonded polyphenols during fermentation, which increases the content of soluble polyphenols and enhances the antioxidant activity *in vitro*.

**Figure 6:**
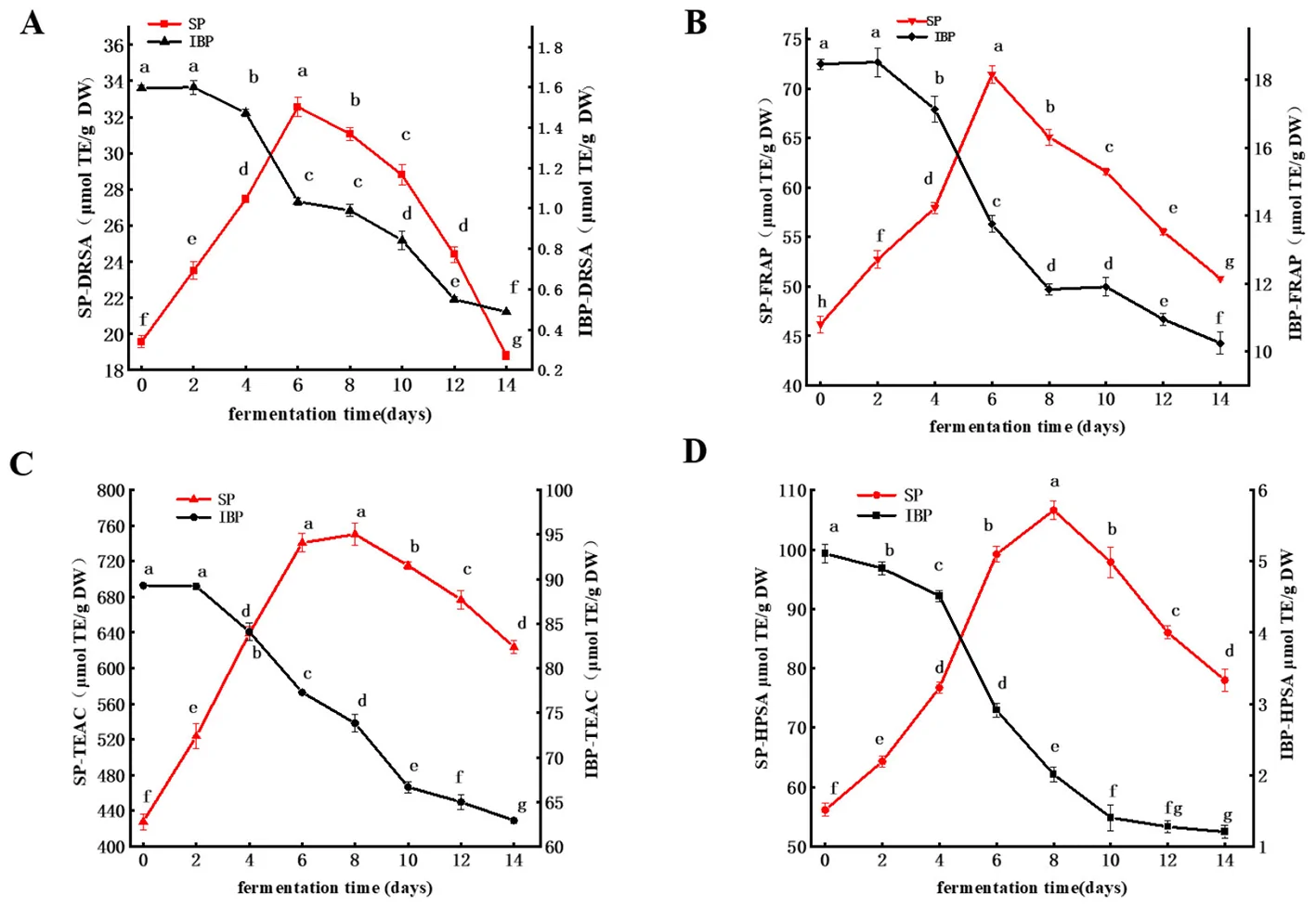
Free radical scavenging activities of phenolic of ML extracts during SSF: **(A)** DRSA, **(B)** FRAP, **(C)** TEAC and **(D)** HPSA assays. SP, soluble polyphenol; IBP, insoluble-bound polyphenol; SP-DRSA, DPPH radical scavenging activity of soluble polyphenol; IBP-DRSA, DPPH radical scavenging activity of insoluble-bound polyphenol; SP-FRAP, ferric reducing antioxidant power of soluble polyphenol; IBP-FRAP, ferric reducing antioxidant power of insoluble-bound polyphenol; SP-TEAC, trolox equivalent antioxidant capacity of soluble polyphenol; IBP-TEAC, trolox equivalent antioxidant capacity of insoluble-bound polyphenol; SP-HPSA, hydrogen peroxide scavenging activity of soluble polyphenol; IBP-HPSA, hydrogen peroxide scavenging activity of insoluble-bound polyphenol. Values represent mean ± SD (*n* = 3). Different letters within each treatment denote a significant difference (*P* < 0.05).

Numerous studies have indicated that correlation matrix analysis is widely used to investigate the relationship between natural bioactive compounds and antioxidant activities (38). In order to analyze the correlation between antioxidant activity and content of polyphenols in the fermentation process of ML, as shown in Figure 3B. The correlation coefficients *R*^2^ of SP content with DRSA, TEAC, FRAP and HPSA were 0.71, 0.93, 0.80 and 0.97, respectively. The results of correlation heat map showed that DRSA, TEAC, FRAP and HPSA were significantly correlated with SP, and their *R*^2^ values were all greater than 0.7 (*P* < 0.05). The correlation coefficients *R*^2^ between the content of IBP and DRSA, TEAC, FRAP and HPSA were 0.97, 0.98, 0.99 and 0.99, respectively. The results indicated that microbial fermentation could increase the content of soluble polyphenols and lead to enhanced activity. The previous studies reported that the capacities of anti-oxidant activity of polyphenols-rich extracts from plant was closely related to the presence of polyphenolic compounds (10, 20)establish the links between phenolic compounds and antioxidant activities of SPs from UF-ML, F-ML extracts, the Pearson correlation analysis was conducted as presented in the figure 7A. The results showed that the antioxidant capacity of F-ML was closely related to the flavan-3-ols content with the correlation between different polyphenols and antioxidant capacity being also significantly different. Therefore, it is likely that different polyphenols contributed differently to the antioxidant capacity of ML. In particular, the (-)-catechin, (-)-epicatechin were strongly positively correlated with the four antioxidant indexes, indicating that these substances were the main contributors to the antioxidant capacity of ML.

**Figure 7:**
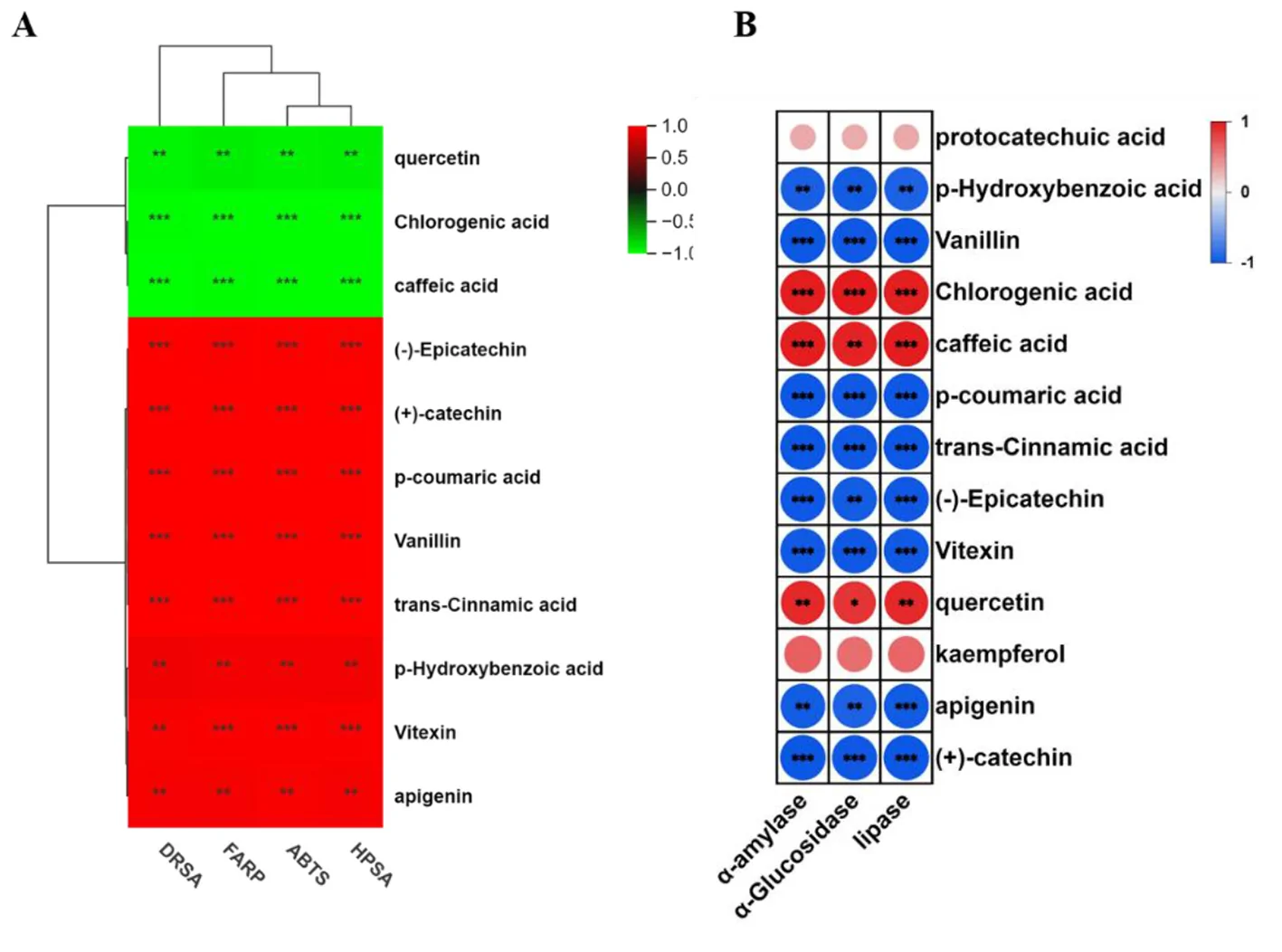
Correlation analysis in ML extracts. **A**. phenolic compounds and antioxidant activities; **B**. phenolic compounds and Inhibitory activities of metabolic syndrome-associated enzymes. The values represent mean ± SD (*n* = 3). *, *P* < 0.05; **, *P* < 0.01; ***, *P* < 0.001.

### 3.6 Metabolic enzyme inhibition

Polyphenols have been reported to excelled in reducing the risk of metabolic syndrome, obesity, diabetes, and other diseases effectively, which the natural carbohydrate hydrolysis inhibitors (44). At present, α-amylase, α-glucosidase and lipase are involved in the inhibition of enzymes involved in the hydrolysis of carbohydrates and fats, which have a positive role in the prevention of related diseases such as diabetes and obesity. The inhibitory effect of the enzyme was characterized by the semi-maximum inhibitory concentration (IC50). The IC50 values of the sample groups were significantly lower than those of the corresponding positive controls (*P* < 0.05) (Table 5), indicating that the ML extracts had a better inhibitory effect on key digestive enzymes. The IC50 values of the ML extracts were expressed as μg GAE/ml, whereas those of the pure reference inhibitors (acarbose and orlistat) were expressed as μg/ml. Because these values are based on different reference units, they are not directly comparable in terms of intrinsic inhibitory potency. Therefore, this difference in the basis of IC50 expression should be considered a limitation of the present comparison. Interestingly, the IC50 value of α-amylase (73.39 ± 0.68 μg GAE/ml), α-glucosidase (88.88 ± 0.25 μg GAE/ml) and lipase (58.82 ± 1.08 μg GAE/ml) in F-ML was significantly lower than that in the UF-ML (*P* < 0.05), which meant that the inhibitory activity of polyphenol extract to the samples was stronger than UF-ML at the same concentration. The results indicated that both F-ML and UF-ML possessed potential natural inhibitory activities against the three enzymes, with the extracts exhibiting a better inhibitory effect than the positive control, suggesting that they may serve a natural enzyme inhibitors for further investigation. Moreover, they may play an important role in the management of metabolic diseases. As illustrated in Figure 7B, the majority of phenolic monomers showed significant correlations with α-amylase, α-glucosidase and lipase activities. Among them, chlorogenic acid, caffeic acid and quercetin were positively correlated with enzyme activities, suggesting that these phenolic substances could act as substrates or intermediate metabolites, participating in the microbial metabolic network and being biotransformation and degradation mechanism of polyphenol components. It was reported that *A. niger* can induce β-glucosidase to hydrolyze 3- glycosidic linkage in isoquercitrin and kaempferol-3-o-glucoside, liberating quercetin and kaempferol (45).

**Table 5:** Determination of α-Amylase, α-Glucosidase and Lipase Inhibitory Activity.

IC_50_	α-Amylase	α-Glucosidase	Lipase
UF–ML	91.44 ± 0.82^b^	96.97 ± 0.88^b^	92.35 ± 2.15^b^
F–ML	73.39 ± 0.68^c^	88.88 ± 0.25^c^	58.82 ± 1.08^c^
acarbose	107.12 ± 1.07^a^	133.25 ± 4.47^a^	
orlistat			138.38 ± 2.39^a^

UF–ML, unfermented moringa leaf; F–ML: fermented moringa leaf. Each value was expressed as the mean ± SD (n = 3). Mean with different small letters (a, b, c) within a row were significantly different (*p* < 0.05).

## 4 Conclusions

This study showed the contribution of *A. niger* to phenolics, antioxidant capacity and metabolic enzyme inhibition of ML. To sum up, we optimized the conditions of SSF with A. *niger* by RSM (fermentation temperature = 30°C, water content = 60%, Inoculation quantity = 10^7^ spores/g, and fermentation time = 8 days). Based on this, cellulase, filter paper, xylanase and β-glucosidase activities occurred with different degrees of improvement after SSF, which might have accelerated the release of IBPs. SPs obtained by SSF exhibited prominent antioxidant, α-amylase, α-glucosidase and lipase inhibitory activities. Moreover, fermentation with *A. niger* significantly increased the content of flavan-3-ols in ML. In particular, the (-)-catechin, (-)-epicatechin were strongly positively correlated with the four antioxidant indexes. The experiment results indicated that the microorganism *A. niger* played a positive regulatory role. Overall, this research has successfully established a SSF method that is beneficial to facilitate the release of IBP from ML exploitation. Moreover, the findings of this research will contribute to the development of polyphenol-based food products or medicines with increased health benefits and antioxidant capacity.

## Data Availability

The original contributions presented in the study are included in the article/supplementary material, further inquiries can be directed to the corresponding authors.
